# Supplementary material for: WordCommentsAnalyzer: A windows software tool for qualitative research
Source: F1000Res. 2018 Sep 4;7:536. Originally published 2018 May 3. [Version 2] doi: 10.12688/f1000research.14819.2 (PMC6137411; doi:10.12688/f1000research.14819.2)
Supplement: Supplementary file 3 [file f1000research-7-17614-s0002.tgz › b2a79496-4ada-47c4-9682-a99ce867836d.zip!/Eight Iranian Health Journals/Journal of Education and Community Health_11.docx]

© Journal of Education and Community Health. This work is licensed under CC BY-NC 4.0 (https://creativecommons.org/licenses/by-nc/4.0/).

Volume & Issue: دوره 2 - شماره 2

Title: تأثیر مشاوره آرام‌سازی پیشرونده عضلانی بر اضطراب زنان نخست باردار مراجعه‌کننده به مراکز بهداشتی درمانی شهر همدان

http://jech.umsha.ac.ir/article-1-118-fa.html

سابقه و هدف: روش آرام‌سازی پیشرونده عضلانی یک روش غیرتهاجمی، کم‌هزینه و بدون عوارض است که به‌طور مستقل توسط خود فرد قابل انجام است. با توجه به اهمیت مسائل روحی روانی زنان در بارداری، هدف از این پژوهش ارزیابی تأثیر مشاوره آرام‌سازی پیشرونده عضلانی بر میزان اضطراب زنان نخست باردار شهر همدان بود. مواد و روش‌ها: پژوهش حاضر یک کارآزمایی بالینی تصادفی بود که بر روی 120 نفر از زنان نخست باردار مراجعه‌کننده به مراکز بهداشتی درمانی شهر همدان در سال1394 انجام گردید. زنان واجد شرایط با استفاده از نمونه‌گیری در دسترس پس از کسب رضایت آگاهانه وارد مطالعه شدند. افراد به صورت تصادفی با در نظر گرفتن پنهان‌سازی اختصاص به دو گروه 60 نفره آزمون و کنترل تقسیم شدند. گروه کنترل فقط مراقبت‌های معمول را دریافت نمودند اما گروه مداخله علاوه بر مراقبت‌های معمول بارداری، پس از دو جلسه مشاوره و فراگیری تکنیک آرام‌سازی عضلانی به مدت 10 هفته، این تمرینات را در منزل انجام دادند و برگه ثبت عملکرد روزانه را تکمیل نمودند. داده‌ها با استفاده از پرسشنامه اطلاعات جمعیت شناختی و پرسشنامه‌ی اضطراب اسپیلبرگر تکمیل گردید. داده‌ها با نرم‌افزار SPSS-20 و آزمون‌های آماری تی‌تست، تی‌زوجی و تحلیل کوواریانس تحلیل شد. یافته‌ها: تحلیل داده‌ها بیانگر همگن بودن سطوح اضطراب آشکار و پنهان در دو گروه قبل از مداخله بود. پس از مداخله در گروه کنترل افزایش معنی‌دار (0.001>P) و در گروه آزمون کاهش معنی‌داری (0.001>P) در میانگین و انحراف معیار اضطراب آشکار و پنهان مشاهده گردید. نتیجه‌گیری: آموزش تکنیک آرام‌سازی پیشرونده عضلانی به زنان باردار می‌تواند تأثیر بسزایی بر کاهش اضطراب آنان داشته باشد.

Title: ساختار عاملی و ویژگی‌های روان‌سنجی پرسشنامه شیوع قُلدری (BPQ) در مدارس متوسطه

http://jech.umsha.ac.ir/article-1-110-fa.html

سابقه و هدف: قلدری پدیده‌ای شایع در مدارس سراسر جهان می‌باشد. بنابراین روا سازی ابزارهای پرکاربرد در این حوزه حائز اهمیت است. هدف از پژوهش حاضر بررسی ساختار عاملی و ویژگی‌های روان‌سنجی پرسشنامه شیوع قلدری بود. مواد و روش‌ها: طرح پژوهش حاضر توصیفی و از نوع مطالعات روان‌سنجی است. تعداد 400 نفر (200 دختر و 200 پسر) از بین دانش‌آموزان مدارس متوسطه شهر قم به روش نمونه‌گیری خوشه‌ای انتخاب شدند و پرسشنامه شیوع قلدری (BPQ) و بهزیستی ذهنی (جهت روایی واگرا) را تکمیل کردند. برای تحلیل داده‌ها از روش‌های تحلیل عاملی، ضریب آلفای کرونباخ و همبستگی پیرسون با استفاده از نرم‌افزارهای SPSS-22 و AMOS-21 استفاده شد. یافته‌ها: نتایج تحلیل عاملی نشان داد که پرسشنامه از سه عامل اشباع شده و از شاخص‌های برازش مناسبی برخوردار است. بین شیوع قلدری با بهزیستی ذهنی (P<0.05, r=-0.10) و پیشرفت تحصیلی (P<0.01, r=-0.18)  رابطه منفی و معنی‌داری مشاهده شد. ضریب آلفای کرونباخ برای کل پرسشنامه 0.81 و برای هر کدام از خرده مقیاس‌ها شامل قلدری 0.81، قربانی 0.75 و نفع اجتماعی 0.70 به دست آمد که  در سطح قابل قبول بودند. نتیجه‌گیری: نسخه فارسی پرسشنامه شیوع قلدری در جامعه دانش‌آموزان، از خصوصیات روان‌سنجی قابل قبولی برخوردار است و می‌توان از آن به عنوان ابزاری معتبر در پژوهش‌های روان‌شناختی استفاده کرد.

Title: نقش سرسختی روان شناختی و سلامت معنوی در تبیین کیفیت زندگی دانشجویان علوم پزشکی کرمانشاه

http://jech.umsha.ac.ir/article-1-138-fa.html

سابقه و هدف: کیفیت زندگی  به عنوان یکی از پدیده ­های مهم سلامتی از عوامل مختلفی تأثیر می پذیرد. پژوهش حاضر با هدف تعیین نقش سرسختی روان­شناختی و سلامت معنوی در تبیین کیفیت زندگی دانشجویان انجام شد. مواد و روش ­ها: طرح پژوهش حاضر توصیفی و از نوع همبستگی بود. جامعه­ آماری این پژوهش شامل کلیه دانشجویان دانشگاه علوم پزشکی کرمانشاه در سال ­تحصیلی 93-1392 بود. حجم نمونه برآورد شده 120 نفر بود که به صورت نمونه ­گیری خوشه ­ای انتخاب شدند. ابزارهای اندازه ­گیری شامل پرسشنامه­ سرسختی روان­شناختی اهواز، مقیاس سلامت معنوی پالوتیزیان و پارک و پرسشنامه کیفیت زندگی (SF-36) و همچنین برگه­ اطلاعات جمعیت ­شناختی دانشجویان بود. تحلیل داده ­ها در ویرایش 19 نرم افزار SPSS و با استفاده از آزمون های آماری ضریب همبستگی پیرسون و تحلیل رگرسیون گام به گام انجام پذیرفت. یافته­ ها: بین سرسختی روان­شناختی و کیفیت زندگی  همبستگی مثبت معنی‌داری وجود داشت (0.05>P). بین سلامت معنوی و کیفیت زندگی نیز رابطه مثبت و معنی‌داری مشاهده شد (0.05>P)، اما بین کیفیت زندگی و سلامت معنوی در بُعد وجودی رابطه معنی‌داری مشاهده نشد، در حالی­که در بُعد مذهبی رابطه معنی‌داری وجود داشت (0.05>P). سرسختی روان­شناختی و سلامت معنوی جمعاً 11.3 درصد واریانس کیفیت زندگی را پیش بینی کردند. نتیجه­ گیری: با توجه به رابطه بین متغیرهای پژوهش، پیشنهاد می‌شود به منظور بالا بردن کیفیت زندگی دانشجویان، با تدارک بسته­های آموزشی در خصوص افزایش سلامت معنوی و سرسختی روان­شناختی در دانشگاه­ ها اقدامات مناسب صورت گیرد.

Title: بررسی تاثیر برنامه آموزشی خودمراقبتی در بهبود کیفیت زندگی سالمندان تحت پوشش مراکز بهداشتی درمانی شهر زنجان

http://jech.umsha.ac.ir/article-1-119-fa.html

سابقه و هدف: سالمندان به علت مشکلات فراوان جسمی و روانی مستعد ابتلا به آسیب های مختلفی می باشند، لذا نیازمند توجه و رعایت برنامه خودمراقبتی موثر در ارتقاء سلامت خود می‌باشند. مطالعه حاضر با هدف بررسی تاثیر مداخله آموزشی برنامه خودمراقبتی بر روند کیفیت زندگی سالمندان انجام گرفت. مواد و روش‌ها: پژوهش حاضر یک مطالعه نیمه تجربی بود که بر روی 160 نفر از سالمندان تحت پوشش 8 مرکز بهداشتی درمانی شهر زنجان انجام شد. نمونه‌ها به روش خوشه‌ای انتخاب شده و به صورت تصادفی به دو گروه آزمون (80 نفر) و کنترل (80 نفر) تقسیم شدند. برای گروه آزمون 6 جلسه آموزشی یک ساعته در زمینه برنامه خود مراقبتی برگزار گردید. ابزارهای مورد استفاده شامل اطلاعات جمعیت شناختی و پرسشنامه کیفیت زندگی SF-36 بود. داده‌های جمع آوری شده با استفاده از نرم افزار SPSS ویرایش 20 و با به کارگیری آزمون‌های کای‌دو، دقیق فیشر، تی‌مستقل و تی‌زوجی مورد تحلیل قرار گرفتند. یافته‌ها: نتایج نشان دهنده افزایش میانگین نمرات ابعاد مختلف کیفیت زندگی سالمندان گروه آزمون پس از انجام مداخله آموزشی بود (0.001>P). نتیجه‌گیری: اگر سالمندان در مورد شیوه‌های صحیح زندگی اطلاعات درستی داشته باشند، می‌توان از بسیاری از مشکلات آنها پیشگیری نمود یا به نحو مطلوبی آنها را کنترل کرده و در جهت ارتقاء کیفیت زندگی آنان برنامه‌ریزی نمود.

Title: نقش استفاده مفرط از تلفن همراه در پیش بینی کیفیت خواب، اضطراب و افسردگی دانشجویان

http://jech.umsha.ac.ir/article-1-130-fa.html

سابقه و هدف: با توجه به توسعه سریع و استفاده گسترده از تلفن همراه و اثرات متنوع آن بر روی تعاملات و ارتباطات، مطالعه اثرات منفی استفاده از تلفن همراه بر روی سلامت کاربران حائز اهمیت است. پژوهش حاضر با هدف تعیین نقش استفاده مفرط از تلفن همراه در پیش بینی کیفیت خواب، اضطراب و افسردگی در بین دانشجویان انجام پذیرفت. مواد و روش‌ها: پژوهش حاضر از نوع توصیفی-همبستگی است. جامعه پژوهش شامل تمامی دانشجویان دانشگاه‌های خوارزمی و پیام نور کرج در سال تحصیلی 94-1393 و نمونه 240 نفر از دانشجویان بود که به روش خوشه ای چند مرحله ای انتخاب گردیدند. برای گردآوری داده‌ها از مقیاس استفاده مفرط از تلفن همراه (COS)، آزمون کیفیت خواب پترزبورگ (PSQI) و مقیاس اضطراب- افسردگی هاسپیتال (HADS) استفاده گردید. تجزیه و تحلیل داده‌ها با استفاده از ویرایش 21 نرم افزار SPSS و به روش همبستگی پیرسون و رگرسیون چندگانه همزمان انجام شد. یافته‌ها: نتایج تحلیل رگرسیون نشان داد که متغیر استفاده مفرط از تلفن همراه، تغییرات کیفیت خواب و اضطراب- افسردگی را به ترتیب با 31 و 14 درصد پیش بینی می‌کند و این متغیر همبستگی مثبت و معنی‌داری و با کیفیت خواب (r=0.56, P<0.001) و اضطراب-افسردگی (r=0.38, P<0.001) دارد. نتیجه‌گیری: استفاده مفرط از تلفن همراه می‌تواند تغییرات کیفیت خواب و اضطراب و افسردگی دانشجویان را پیش‌بینی نماید و پژوهشگران و متخصصین روان درمانی می‌توانند با کنترل این عامل، مسبب ارتقاء کیفیت زندگی و سلامت روان دانشجویان گردند.

Title: تأثیر آموزش پیشگیری از پوکی استخوان بر آگاهی، نگرش و رفتار دانش‎آموزان دختر نوجوان شهر کرمانشاه

http://jech.umsha.ac.ir/article-1-74-fa.html

سابقه و هدف: پوکی ­استخوان یک اختلال متابولیک استخوانی شایع در زنان به شمار می‌رود. آسان­ ترین و ارزان­ ترین راه مقابله با این بیماری پیشگیری اولیه است، لذا آموزش عوامل خطر و رفتارهای پیشگیرانه در سال­های نوجوانی اهمیت زیادی دارد. پژوهش حاضر با هدف تعیین تاثیر آموزش پیشگیری از پوکی ­استخوان بر آگاهی، نگرش و رفتار دانش‎آموزان دختر در کرمانشاه صورت گرفت. مواد و روش‌ها: مداخله ‌نیمه­ تجربی حاضر بر روی 118 دانش ­آموز دختر شهر کرمانشاه در سال 1393 انجام شد. شرکت کنندگان با نمونه ­گیری خوشه ­ای از مدارس راهنمایی ناحیه سه شهر انتخاب شدند و به‎صورت تصادفی در دو گروه آزمون (53 نفر) و کنترل (65 نفر) قرار گرفتند. ابزار گردآوری اطلاعات، پرسشنامه ه­ای چهاربخشی شامل مشخصات جمعیت‌شناختی، آگاهی، نگرش و رفتار در زمینه پیشگیری از پوکی­استخوان بود. داده‌ها قبل و یک ماه بعد از مداخله­ آموزشی با استفاده از نرم‎افزار SPSS-19 و آزمون‌های تی‌مستقل، تی‌زوجی، کای دو و همبستگی پیرسون مورد تجزیه و تحلیل قرار گرفتند. یافته‌ها: میانگین نمره آگاهی، نگرش و رفتار در گروه آزمون و کنترل در قبل از مداخله تفاوت معنی‌دار نداشت (0.05<P). پس از اجرای برنامه آموزشی، میانگین نمره آگاهی (0.024=P) و نگرش (0.003=P) افزایش معنی‌داری در افراد گروه آزمون نسبت به گروه کنترل نشان داد، اما میانگین نمره رفتار در دو گروه اختلاف معنی‌داری نداشت (0.086=P). نتیجه‎گیری: آموزش در زمینه پیشگیری از پوکی ‎استخوان باعث افزایش آگاهی و نگرش دانش­ آموزان دختر در کرمانشاه گردید ولی منجر به تغییر رفتار نگردید که بایستی این مساله در طراحی مداخلات آموزشی در این گروه هدف مدنظر باشد.

Title: اثربخشی برنامه آموزشی مبتنی بر نظریه رفتار برنامه ریزی شده بر افزایش مصرف صبحانه در بین دانش آموزان دبیرستانی شهر همدان

http://jech.umsha.ac.ir/article-1-100-fa.html

سابقه و هدف: صبحانه اولین وعده غذای مصرفی در روز است و مصرف آن توسط کودکان و نوجوانان با دریافت کافی مواد مغذی ارتباط دارد. مطالعه حاضر با هدف تعیین اثربخشی برنامه آموزشی مبتنی بر نظریه رفتار برنامه ریزی شده در افزایش مصرف صبحانه در بین دانش آموزان دبیرستانی شهر همدان انجام گردید. مواد و روش ها: این مطالعه نیمه تجربی بر روی 140 نفر (70 نفرگروه مداخله و 70 نفر گروه کنترل) از دانش آموزان دبیرستانی شهر همدان که به روش نمونه‌گیری تصادفی ساده انتخاب شدند، انجام شد. پرسشنامه مشتمل بر سوالات دموگرافیک و سازه های نظریه رفتار برنامه ریزی شده بود که به روش خودگزارش دهی قبل و دو ماه بعد از مداخله آموزش توسط دانش آموزان گروه مداخله و کنترل تکمیل گردید. شرکت کنندگان گروه مداخله سه جلسه آموزشی مبتنی بر سازه های نظریه رفتار برنامه ریزی شده را دریافت کردند. داده ها با استفاده از SPSS-19 و با آزمون های تی مستقل، تی زوجی و کای دو مورد تجزیه و تحلیل قرار گرفت. یافته ها: یافته های پژوهش نشان داد که بین میانگین نمره مربوط به سازه های تئوری رفتار برنامه ریزی شده (نگرش، کنترل رفتاری، قصد رفتاری) در گروه مداخله قبل و بعد از مداخله آموزشی اختلاف معنی داری وجود دارد. نتیجه گیری: با توجه به نتایج حاصل از این مطالعه، لازم است که برنامه های آموزشی مناسب جهت افزایش مصرف صبحانه در مدت زمان طولانی تری با استفاده از سایر تئوری های آموزش انجام شود.
